# Does segmental artery occlusion cause intravertebral cleft following osteoporotic vertebral fracture: a prospective magnetic resonance angiography study

**DOI:** 10.1186/s12891-022-05064-8

**Published:** 2022-01-31

**Authors:** Tianyu Zhang, Yu Kang, Yanhua Wang, Peixun Zhang, Dianying Zhang, Feng Xue

**Affiliations:** 1 Department of Urology, China-Japan Friendship Hospital, Beijing, 100029 China; 2 Department of Radiology, Peking University People’s Hospital, Beijing, 100044 China; 3 Department of Traumatic Orthopaedics, Peking University People’s Hospital, Beijing, 100044 China; 4 Institute of Trauma and Nerve Regeneration, Peking University People’s Hospital, Beijing, 100044 China

**Keywords:** Intravertebral cleft, Osteoporosis vertebral compression fracture, Magnetic resonance angiography, Segmental artery occlusion

## Abstract

**Background:**

The avascular necrosis (AVN) hypothesis of intravertebral cleft (IVC) formation in osteoporotic vertebral fracture (OVCF) has received increasing attention. The aim of this article is to detect whether the segmental artery occlusion causes the IVC following OVCF.

**Methods:**

Between December 2019 and April 2020, 44 OVCF patients with 46 fracture levels were prospectively enrolled and the vertebral segmental arteries were evaluated by magnetic resonance angiography (MRA). The artery conditions were divided into patent, narrow and occluded. The lesion segmental occlusion rate (LSOR) and the total occlusion rate (TOR) were calculated. The association of segmental artery occlusion and IVC formation was assessed.

**Results:**

LOSR was 15.34% and TOR was 15.12%. The segmental arteries of the unfractured vertebrae had a higher occlusion rate at thoracolumbar levels than at non-thoracolumbar levels. There was no significant difference between the IVC group and the non-IVC group in the fractured levels artery occlusion rate (20.24 ± 28.08 vs 9.78 ± 19.56, *P* = 0.156) or the total segmental arteries occlusion rate (13.83 ± 12.04 vs 11.57 ± 9.25, *P* = 0.476).

**Conclusions:**

In patients with vertebral osteoporotic fracture, segmental artery occlusion is not associated with the development of intravertebral cleft.

## Introduction

**Fig. 1:**
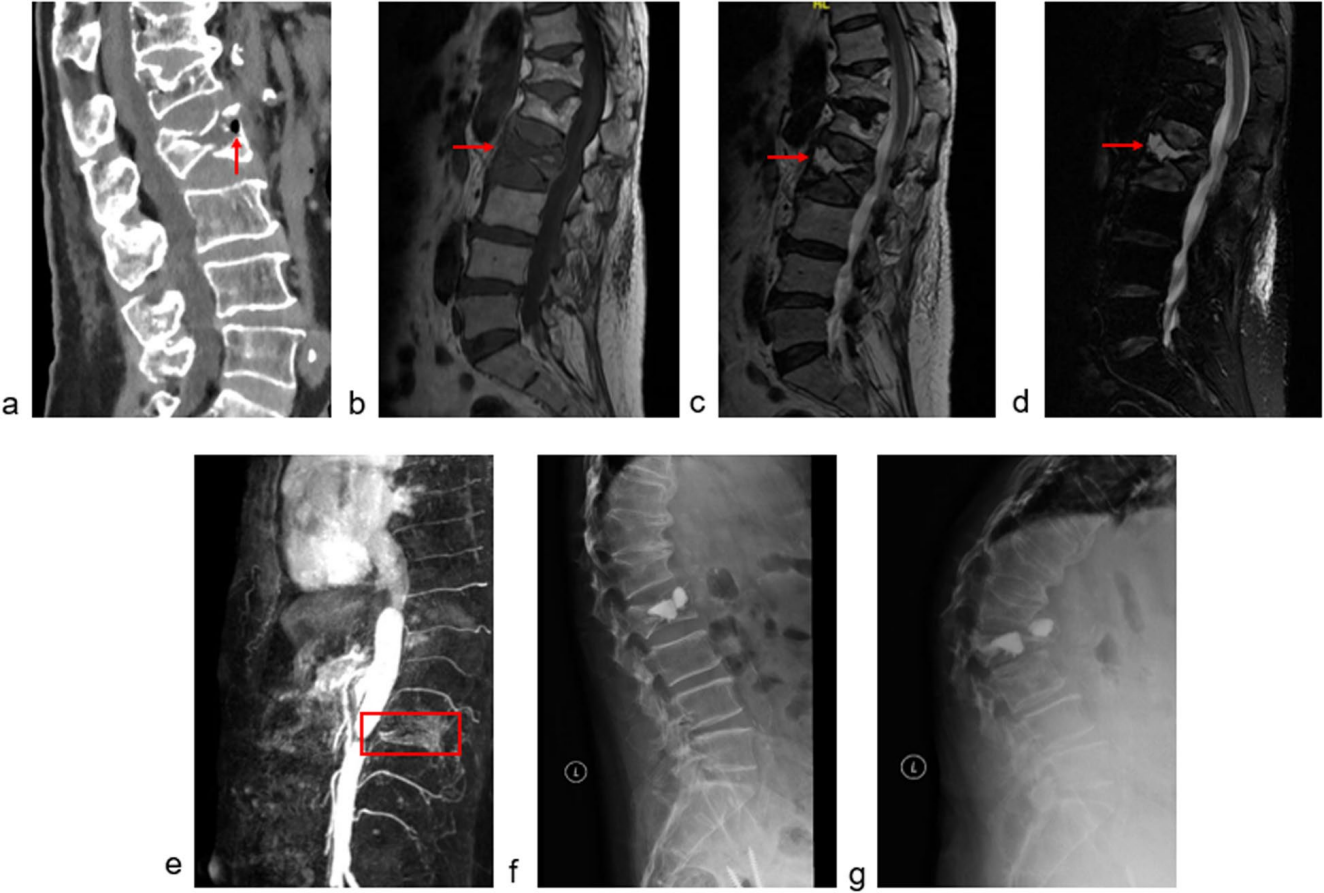
An eighty-two-year-old female suffered from severe back pain for about 17 days (VAS 9) after left heavy object. CT showed an L2 compression fracture with intravertebral cleft formation (**a**) and MRI indicated an area of hypointensity on T1 and hyperintensity on T2 and T2-fat suppression images (**b**, **c**, **d**, red arrow). MRA revealed that both sides of the L2 segmental arteries were occluded (**e**). Unilateral vertebroplasty was performed for the patients (**f**). The augmented vertebra collapsed and the cement was displaced half a year after the procedure (**g**)

Osteoporosis vertebral compression fracture (OVCF) is the most common osteoporosis fracture. Although conservative or minimally invasive treatment can always relieve pain, poor prognosis frequently occurs in OVCF patients combined with intravertebral cleft (IVC) [1, 2]. IVC is the classic radiological sign of Kummell’s disease [3], which is an area of low-intensity on T1-weighted and high-intensity or low-intensity on T2-weighted magnetic resonance images (MRI) in fracture vertebrae (Fig. 1b, c, d) [3]. The incidence of complications such as cement leakage [4], augmented vertebral recollapse (Fig. 1f, g) [5], and adjacent vertebral fracture [6] was high when OVCFs were combined with IVCs. Therefore, exploring IVC pathogenesis is important for preventing and finding better treatment methods.

The hypotheses of IVC formation are controversial, including the avascular necrosis (AVN) hypothesis [7], air formation hypothesis [8], biomechanical alteration hypothesis [9], and pseudarthrosis formation hypothesis [10]. The AVN hypothesis received much attention among the above hypotheses. IVC is a sign of osteonecrosis caused by the destruction of blood supply [11, 12]. There is growing evidence supporting this idea. Dupuy et al. [13] and Libicher et al. [7] both revealed that the pathological characteristic of IVC was avascular osteonecrosis by biopsy. Once vertebral compression fractures occur, the vertebrae require more blood supply for osteocytes growth and vertebral reconstruction [14]. If arteries cannot provide sufficient blood supply, osteogenesis might arrest, and vertebrae nonunion occur. Lin et al. [11] found that the IVC was related to poor perfusion of the adjacent vertebral bone marrow by dynamic contrast-enhanced magnetic resonance imaging. Kim et al. [12] found a high artery occlusion rate in IVC patients by magnetic resonance angiography (MRA). However, due to the lack of a control group in Kim’s study, segmental artery occlusion leading to the IVC could not be concluded. Therefore, we hypothesized that IVC patients would have a higher segmental artery occlusion rate than non-IVC patients. The aim of this study was to elucidate the association between segmental artery occlusion and IVC formation (Fig. 1e).

## Methods

The study was approved by the Ethics Review Committee of Peking University People’s Hospital with approval number 2019PHB240. All the written informed consents were acquired from the patients.

### Patients

We prospectively recruited 44 patients (male: female = 14: 30) with OVCFs from the traumatic orthopedic department at a tertiary referral center. Recruitment was started in December 2019 with the completion of enrollment in April 2020. The inclusion criteria were as follows: 1) patients with osteoporosis vertebral compression fracture; 2) back pain over 1 week prior to starting intervention; 3) focal tenderness on the OVCF level; and 4) scheduled to receive vertebroplasty surgery. The exclusion criteria included 1) vertebral fracture caused by infection or malignancy; 2) neurological impairment; and 3) MRA contraindication. All patients underwent X-ray and MRA for the thoracic and/or lumbar spine during the first hospitalization.

### Sample size calculation

For comparison of the segmental artery occlusion rate between the non-IVC group and IVC group, we calculated the sample size according to a previous study. A previous study revealed that the occlusion rate of the segmental artery in patients with IVC was 57.8% [12]. The incidence of segmental artery occlusion in non-IVC patients was 19–27% [15]. Therefore, we assumed that the incidence of artery occlusion in the IVC group was 60% and that the segmental artery occlusion rate in the non-IVC patient was 30%. The α was 0.05 and β was 0.2. The number of vertebral level in the IVC group and the non-IVC group was 1:1. The calculated sample size was as follows: the number of arteries in the non-IVC group was 40, and the number of arteries in the IVC group was 40 [16].

### Data collection

Basic characteristics of the patients, including age, gender, body mass index (BMI), time from injury to MRI diagnosis, and fracture levels, were collected from the clinical files. Vertebral avascular risk factors were recorded to reflect the blood supply condition [17]. The number of factors including hypertension, diabetes mellitus, coronary heart disease, cerebral infarction, hormone usage, and smoking was regarded as the vertebral avascular risk factor score.

Imaging data including the X-ray or CT, MRI, and MRA results, were reviewed by the double-blind method. The IVC diagnosis and vertebral occlusion condition were separately assessed by two experienced surgeons. The compression rate (CR) was calculated according to previous study [4]. Fracture severity was graded as grade 1: mild (< 25% collapse); grade 2: moderate to severe (> 25% collapse) [5]. The diagnosis of IVC was based on the imaging sign of low-intensity on T1-weighted area and high-intensity or low-intensity area on T2-weighted MRI in fracture vertebra (Fig. 1).

**Fig. 2:**
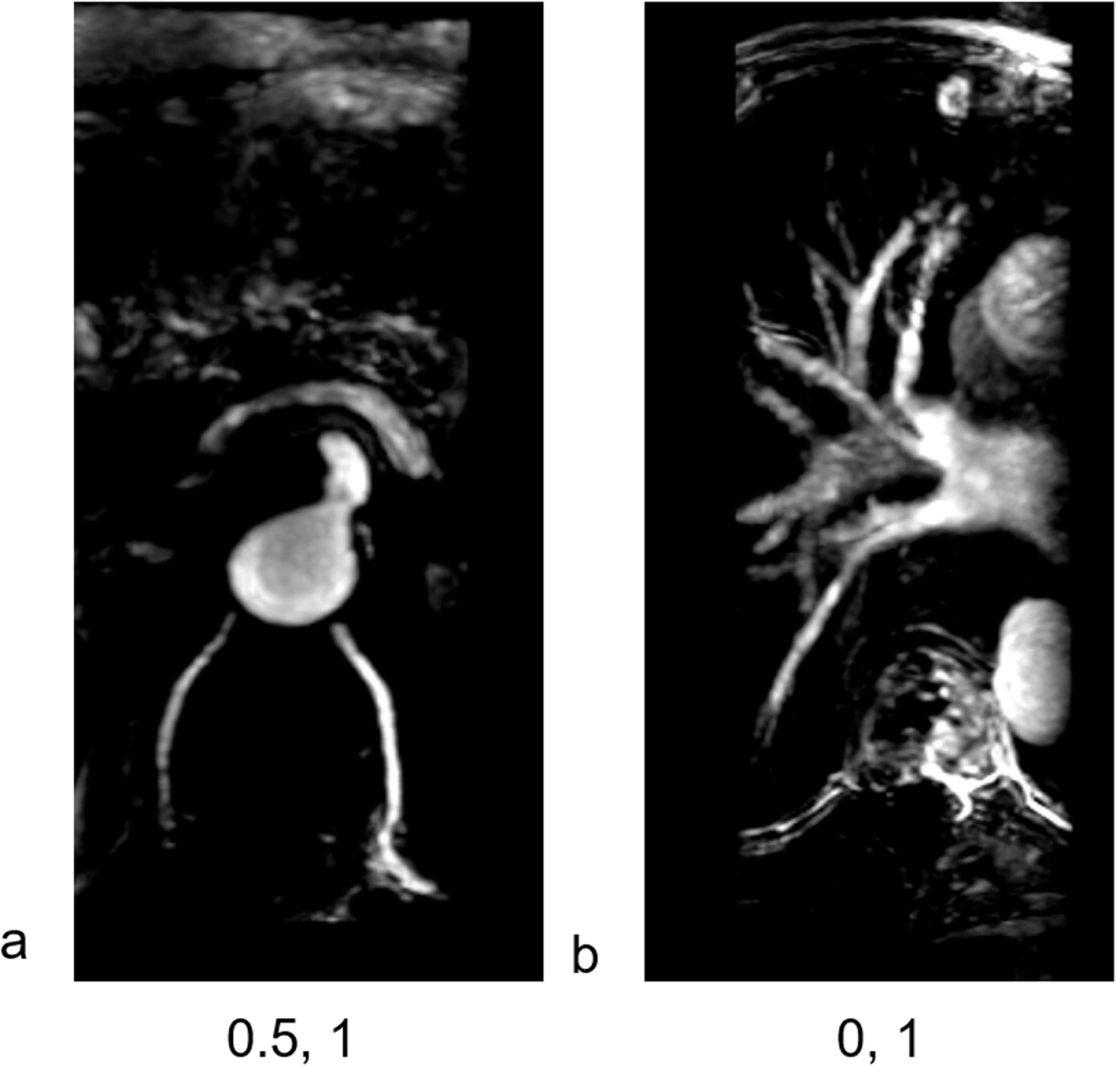
Each segmental artery condition was classified into patent, narrow and occluded and was scored as 1, 0.5, and 0, respectively

According to the MRA image, each segmental artery condition was classified into patent, narrow and occluded and was scored as 1, 0.5, and 0, respectively (Fig. 2). The lesion segmental occlusion rate (LSOR) and the total occlusion rate (TOR) were calculated. The LOSR reflected the fracture level blood supply. LOSR = (2 − fracture level segmental artery occlusion condition score)/2 × 100%. Except for L5, segmental arteries of each thoracic and lumbar vertebrae were in pairs [18]. Therefore, the L5 level was not included in the LOSR calculation. The TOR reflected the total spine blood supply. All MRAs covered T10 to L4 segments, and the corresponding artery conditions were recorded; therefore, the TOR was calculated using data from T10 to L4.

$$\mathrm{TOR}=\left(14-\sum_{L4}^{T10} segmental\ artery\ occlusion\ condition\ score\right)/14\times 100\%$$

The compression vertebral segmental artery occlusion condition was assessed by an experienced surgeon and repeated with an interval of more than 1 month. The intraclass correlation efficiency in segmental artery occlusion was 0.788 (*P* < 0.001).

### Data analysis

Patients were classified into the IVC group and the non-IVC group according to the diagnosis criteria. Age, gender, BMI, vertebral avascular risk factors, thoracolumbar levels (T11-L2), fracture severity, compression ratio, time from injury to MRI, LSOR and TOR were compared between the IVC and non-IVC groups.

### Statistical analysis

Univariate analysis was analysed using SPSS Ver. 22.0 for Windows (IBM Corp. NY, USA). Continuous variables were analysed with chi-square tests and are presented as the mean ± standard deviation. Categorical variables were analysed with Mann-Whitney U tests and are presented as the median (IQR). The dichotomic variables were analysed with the Student’s t-tests and presented as numbers (%). *P* <0.05 was regarded as a significant difference.

## Results

**Fig. 3:**
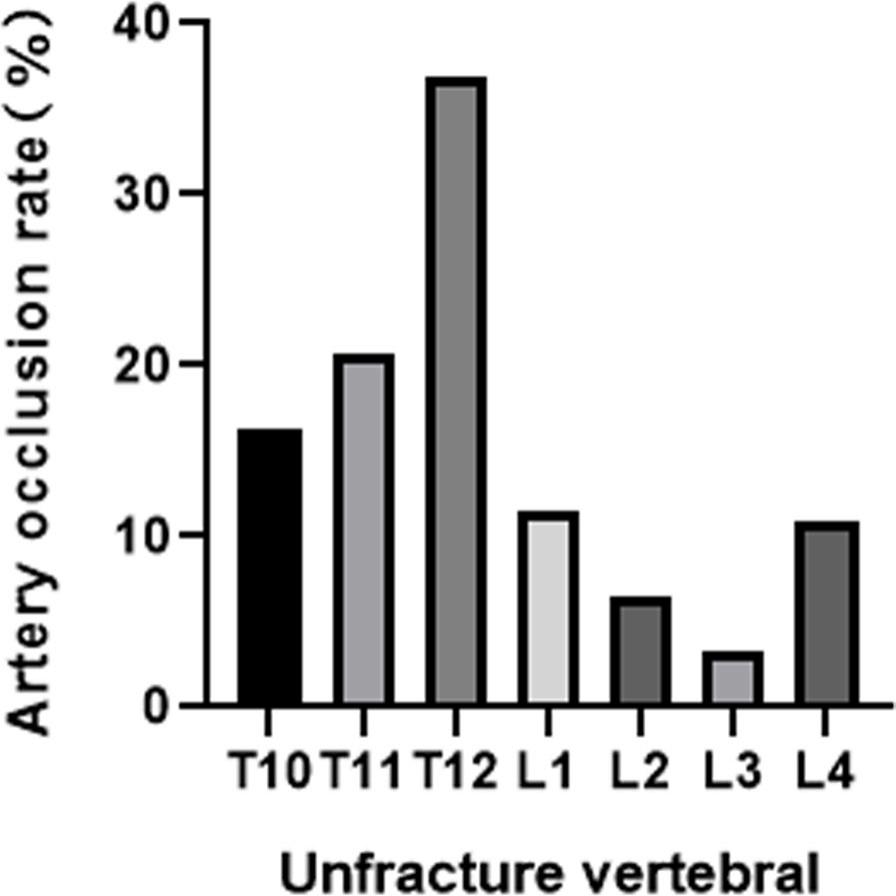
Occlusion rate of each unfractured vertebral segmental artery

A total of 44 patients (male: female = 14: 30) with 46 fractured vertebrae and 92 vertebral segmental arteries were included in this study. The mean age of the patients was 74.6 ± 12.38 years old. Twenty-one (45.6%) compression vertebrae had IVC. The time from injury to MRI was 21 ± 44.5 days (Table 1). A total of 588 vertebral arteries were analysed by MRA. The total segmental artery occlusion rate was 15.34%. Among the unfractured levels, artery occlusion or narrow rate of T10 was 20.9%, T11 was 23.8%, T12 was 34.3%, L1 was 23.3%, L2 was 8.4%, L3 was 4.6%, and L4 was 8.9% (Fig. 3).

**Table 1:** Characteristics of Patients

Variable	Patients number (*n*=44)
Age (years)	74.6 ±12.38
Gender (female)	30 (68.2%)
BMI (kg/m^2^)	23.19 ± 3.04
fracture severity (>25% collapse)	20 (44.4%)
CR (%)	79 ± 18
Time from injury to MRI (Days)	21 ± 44.5
Thoracolumbar levels (T11-L2)	34 ± 77.3
LSOR (%)	15.12
TOR (%)	15.34

*n* patient number, *BMI* Body mass index, *CR* Compression rate, *LSOR* Lesion segmental occlusion rate, *TOR* Total occlusion rate

LOSR = (2 − fracture level segmental artery occlusion condition score)/2 × 100 % )

$$\mathrm{TOR}=\left(14-\sum_{L4}^{T10} segmental\ artery\ occlusion\ condition\ score\right)/14\times 100\%$$.

The fractured vertebrae were T5 in 1 patient, T9 in 4 patients, T10 in 1 patient, T11 in 3 patients, T12 in 9 patients, L1 in 14 patients, L2 in 8 patients, L3 in 1 patient, L4 in 3 patients, and L5 in 2 patients. Among these, 42 vertebral segmental arteries were included in the IVC group and 50 in the non-IVC group. The time from injury to MRI was significantly different between the IVC group and the non-IVC group (39.69 ± 64.49 vs. 9.07 ± 12.18, *P* = 0.041). There was no significant difference in age, gender, BMI, vertebral avascular risk factors, thoracolumbar levels (T11-L2), fracture severity, or compression ratio (*P* > 0.05). Neither the lesion segmental occlusion rate (LSOR) nor the total occlusion rate (TOR) showed significant difference between the IVC group and the non-IVC group (LSOR 20.24 ± 28.08% vs. 9.78 ± 19.56%, TOR 13.83 ± 12.04% vs. 11.57 ± 9.25%, *P* > 0.05) (Table 2).

**Table 2:** Comparison of the IVC group with non-IVC group

Factors	IVC (*n*=21)	Non-IVC (*n*=25)	*P* values
Age (years)	76.48 ± 12.46	72.64 ± 12.14	0.297
Gender (female)	15 (71.4%)	17 (68.0%)	0.801
BMI (kg/m^2^)	22.72 ± 3.10	22.34 ± 2.94	0.486
Vertebral avascular risk factors	1 (2)	1 (2)	0.563
Thoracolumbar levels (T11-L2)	17 (81.0%)	17 (68.0%)	0.319
Fracture severity (>25%)	10 (47.6%)	9 (36.0%)	0.425
CR (%)	77.94 (20.26)	81.11 (16.83)	0.861
Time from injury to MRI (Days)	39.69 ± 64.49	9.07 ± 12.18	0.041*
LSOR (%)^a^	20.24 ± 28.08	9.78 ± 19.56	0.156
TOR (%)	13.83 ± 12.04	11.57 ± 9.25	0.476

*P<0.05. Data number (%) are for fracture severity, CR and gender; median (IQR) for vertebral avascular risk factors; mean ± SD for other variables. n= fracture number, ^a^ Two L5 segmental fracture was not included in LSOR calculation. *BMI* Body mass index, *CR* Compression rate, *MRI* Magnetic resonance image, *LSOR* Lesion segmental occlusion rate, *TOR* Total occlusion ratio

## Discussion

IVC formation in OVCFs was significantly associated with poor prognosis [4–6]. However, the pathogenesis of IVC is still controversial. Whether segmental artery occlusion causes IVC has received much attention.

In this study, the LOSR and TOR were not significant different between the IVC group and the non-IVC group. LOSR reflected the local vertebral blood supply, and TOR reflected the whole vertebral blood supply. This study indicated that vertebral segmental artery occlusion was not associated with IVC formation.

**Fig. 4:**
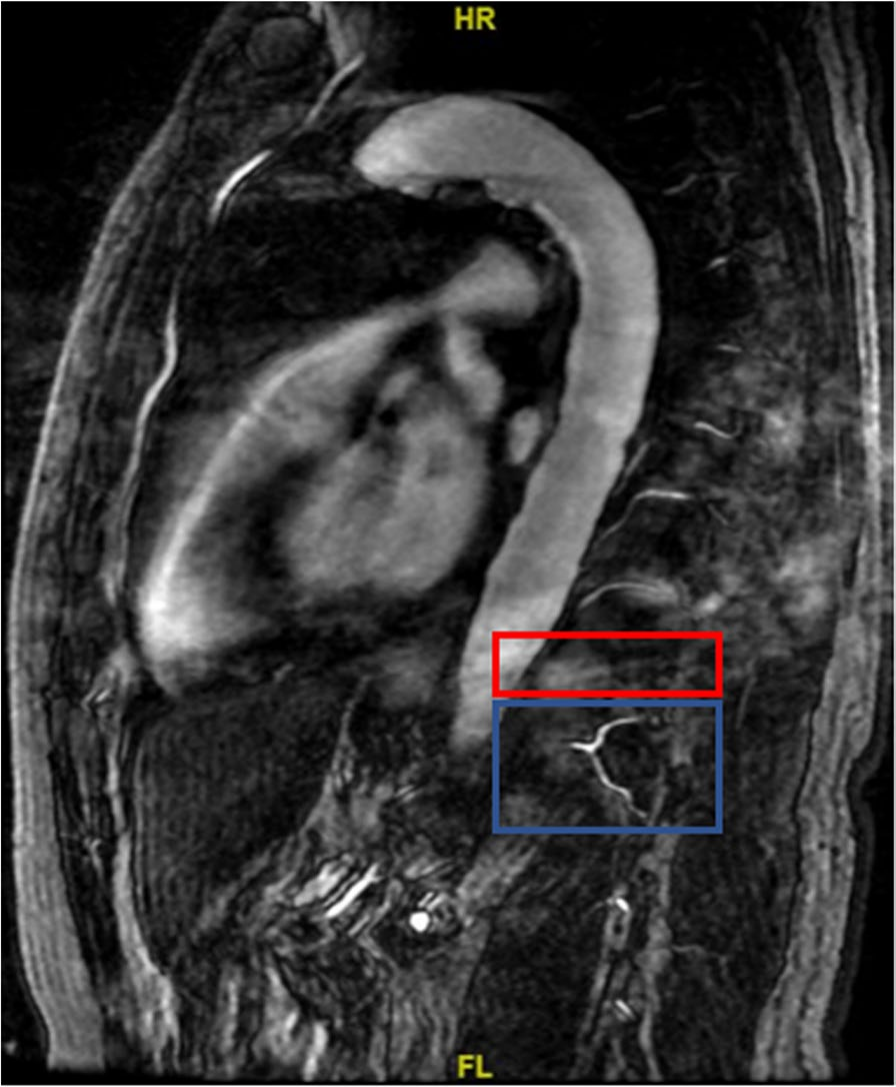
Adjacent segmental artery formed collateral circulation (blue) to compensate for the ischemic level (red)

In a previous study, Nambu et al. [19] showed that bilateral segmental artery ligation reduced vertebral blood perfusion. Lin et al. [11] found that adjacent vertebral marrow poor blood perfusion was associated with IVC formation. These two studies indicated causality between segmental artery occlusion and IVC. However, we concluded the contrary result. Collateral arteries formed to compensate for the vertebral blood supply when the segmental artery was occluded [18] (Fig. 4). Therefore, artery occlusion might not completely reflect the vertebral blood supply and could not be used for predicting IVC formation.

From T10 to L4, there were pairs of segmental arteries of the vertebrae. The L5 segmental artery was the median sacral artery, which was not included in the LOSR assessment [20]. We assessed the segmental artery condition from T10 to L4 and found that segmental artery occlusion was more common in the thoracic-lumbar region. The thoracic-lumbar region is a transition zone from the thoracic region to the lumbar region that has the maximum motion range and sufferes much stress [21]. These two factors make the thoracic-lumbar region arteries prone to occlusion. Kim et al. [12] proposed that the vertebral fracture caused segmental arteries occlusion. In our study, the occlusion rate of the fracture levels was the same as the total segmental artery occlusion rate. This result indicated that vertebral fracture was not associated with segmental artery occlusion.

There were several limitations in this study. This was not a large-scale study. However, we calculated the sample size according to previous studies and considered that the sample size was enough to draw the conclusion. Another weakness was that the detection of IVC and artery occlusion was at the same time point. Hence, the influence of vertebral long-term ischemia could not be assessed. Artery occlusion without IVC formation did not indicate that IVC would not form after the MRA examination. Repeated MRI evaluations might help to further investigate the association between the IVC and artery occlusion. Because the patients included in our study received the vertebroplasty surgery, IVC formation was impossible to recheck.

## Conclusion

This study provided evidence that segmental artery occlusion did not lead to the IVC. Vertebral compression fracture was not associated with segmental artery occlusion.

## Data Availability

The datasets generated and analyzed during the current study are not publicly available due to the data also forms part of an ongoing study but are available from the corresponding author on reasonable request.

## Abbreviations

OVCF: Osteoporosis vertebral compression fracture
IVC: Intravertebral cleft
MRI: Magnetic resonance image
AVN: Avascular necrosis
MRA: Magnetic resonance angiography
BMI: Body mass index
CR: Compression rate
LSOR: Lesion segmental occlusion rate
TOR: The total occlusion rate
